# Differences among canine serum, plasma and urine metabolite profiles from samples that were collected at the same time

**DOI:** 10.3389/fvets.2026.1837846

**Published:** 2026-05-21

**Authors:** Robin Moore, Johanna Anturaniemi, Vidya Velagapudi, Anna Hielm-Björkman

**Affiliations:** 1 Department of Equine and Small Animal Medicine, Faculty of Veterinary Medicine, University of Helsinki, Helsinki, Finland; 2 Metabolomics Unit, Institute for Molecular Medicine Finland FIMM, University of Helsinki, Helsinki, Finland

**Keywords:** canine, metabolomics, plasma, serum, urine

## Abstract

**Background:**

Biospecimen choice can substantially influence metabolite measurements, yet matched comparisons of serum, EDTA plasma, lithium-heparin plasma, and urine remain limited in canine targeted metabolomics.

**Objectives:**

To evaluate cross-matrix comparability of targeted small polar metabolite profiles in dogs and to assess how different biospecimens capture diet- and health-associated variation.

**Animals:**

Client-owned dogs from a previously reported feeding trial.

**Methods:**

Targeted ultra-performance liquid chromatography–tandem mass spectrometry (UPLC-MS/MS) was performed on matched biospecimens from a baseline blood-matrix cohort (serum, EDTA plasma, lithium-heparin plasma) and an end-of-trial serum-urine cohort. Metabolites with >20% missing values within a matrix and samples with >20% missing metabolite values within a biofluid were excluded. Cross-matrix agreement was assessed using Pearson correlation and mean paired log_10_ concentration differences. PCA and PERMANOVA were used to compare global matrix effects and diet- and health-associated separation.

**Results:**

After filtering, 88 metabolites remained for serum, 85 for EDTA plasma, and 88 for lithium-heparin plasma in the blood-matrix cohort. Globally, EDTA plasma separated clearly from both serum and lithium-heparin plasma, whereas serum and lithium-heparin plasma overlapped substantially. At the metabolite level, 10/84 (11.9%) serum–EDTA plasma, 16/88 (18.2%) serum–lithium-heparin plasma, 20/84 (23.8%) lithium-heparin plasma–EDTA plasma, and 4/75 (5.3%) serum–urine comparisons were comparable to varying degrees. Serum showed the clearest diet-associated separation at baseline (PERMANOVA pseudo-*R*^2^ = 0.13, *p* = 0.013) and end-of-trial (pseudo-*R*^2^ = 0.22, *p* = 0.021). Health-associated separation was weak and exploratory.

**Conclusions and Clinical Importance:**

Biospecimen choice materially affects targeted metabolite profiling in dogs. Serum and lithium-heparin plasma were globally more similar to each other than either was to EDTA plasma, whereas urine was a distinct complementary matrix rather than a blood surrogate. Serum was the most robust matrix for detecting diet-associated variation.

## 1 Introduction

High-quality quantitative metabolomics depends on careful control of pre-analytical and matrix-related variation. Differences in matrix composition, protein content, enzymatic activity, ion chelation, and sample processing can introduce non-biological variation that affects metabolite detectability, quantitative reliability, and the interpretability of downstream statistical analyses. In both human and veterinary metabolomics, biospecimen choice is therefore a core methodological decision rather than a neutral sampling detail.

This is particularly important when comparing serum and plasma. In serum, the clotting period before separation allows ongoing cellular metabolism, platelet activation, and proteolysis, which can shift concentrations of small polar metabolites relative to plasma (1, 2). In EDTA (ethylenediaminetetraacetic acid) plasma, chelation of Ca^2^^+^ and Mg^2^^+^ suppresses clotting and some enzyme activity, but can also influence specific analytes, including amino acids and biogenic amines (for example, arginine and taurine), as well as acylcarnitines, nucleotides, trimethylamine-N-oxide, and sarcosine (2–7). Lithium-heparin plasma acts through the antithrombin pathway without strong chelation (8). Despite these known differences between plasma types, many studies describing plasma–serum comparisons do not clearly specify the anticoagulant used for plasma collection. Even targeted metabolomics studies using standardized kits often treat plasma as a single category while relying specifically on EDTA plasma (7). This limits cross-study comparability and weakens the generalizability of reported plasma–serum differences.

EDTA plasma and serum often track closely for many amino acids, but their concentrations are not necessarily interchangeable (3), and lithium-heparin plasma has in some comparative datasets shown fewer differences from serum overall (8). Biogenic amines may be particularly sensitive to clotting-related effects, platelet or cellular activity, handling time, and low-abundance quality-control fragility (2). Prior blood-metabolomics studies suggest that acylcarnitine behavior is metabolite-specific: some medium- and long-chain acylcarnitines show little difference between serum and EDTA plasma, whereas others differ systematically between serum and plasma (1, 2). For glucose-related measures, pre-analytical delay remains critical because glycolysis continues until separation and cooling, and neither EDTA nor lithium-heparin fully eliminates this issue (2).

Urine represents a fundamentally different analytical matrix. Unlike blood, it is not homeostatically regulated and is strongly influenced by hydration, renal handling, diet, and the microbiome (9). Meaningful comparisons therefore require normalization, for example to creatinine, osmolality, specific gravity, or robust alternatives (9). Urine can nevertheless be highly informative for small polar metabolites, particularly diet-derived compounds, microbial co-metabolites, and other excretion products, and it offers the practical advantage of non-invasive sampling. At the same time, urinary amino acids, biogenic amines, and acylcarnitines often show higher variance and weaker direct correspondence to circulating concentrations than their blood-based counterparts (10, 11).

In companion dogs, metabolomics has already been applied to diverse clinical questions, including idiopathic inflammatory bowel disease, acute diarrhea, and urogenital cancer (12). However, systematic within-individual comparisons of matched serum, EDTA plasma, lithium-heparin plasma, and urine remain scarce in veterinary medicine. To our knowledge, no such data have been reported in dogs. Studies in humans indicate that biospecimen choice and pre-analytical handling influence the measurement of amino acids, energy-related metabolites including tricarboxylic acid intermediates, nucleotides, peptides, and lipid-related metabolites, but equivalent evidence in dogs remains limited (2, 13).

An additional strength of the present study is that the analyzed biospecimens were derived from a previously reported feeding trial in client-owned dogs that evaluated both dietary exposure and clinical phenotype (14). This design makes it possible not only to assess analytical agreement across matrices, but also to compare how different biofluids capture biologically relevant variation. The matched sample set can therefore be used to evaluate both methodological interchangeability and biological sensitivity across serum, EDTA plasma, lithium-heparin plasma, and urine.

Given the increasing use of quantitative targeted liquid chromatography–mass spectrometry (LC-MS) platforms in veterinary research and diagnostics, there is a need for practical guidance on which metabolites can be compared reliably across matrices, which remain matrix-specific, and which biofluids are best suited to capture biological effects. In the present study, we performed targeted ultra-performance liquid chromatography–tandem mass spectrometry (UPLC-MS/MS) metabolomics on matched serum, EDTA plasma, lithium-heparin plasma, and urine samples from client-owned dogs. Our aims were to determine which metabolites and metabolite classes show stable cross-matrix behavior, to identify matrix-specific differences, and to provide guidance for selecting the most appropriate biofluid for analysis of small polar non-ionic metabolites in canine studies.

## 2 Materials and methods

### 2.1 Animals

Client-owned dogs were enrolled into two matched-sample cohorts collected at two time points of a previously reported diet trial, baseline (blood-matrix cohort) and end of trial (serum-urine cohort) (14–17). In the blood-matrix cohort, serum, EDTA plasma, and lithium-heparin plasma were collected simultaneously from 11 dogs (Staffordshire bull terrier, *n* = 8; Australian shepherd, *n* = 1; Labrador retriever, *n* = 1; mixed-breed, *n* = 1), of which eight Staffordshire bull terriers continued and completed the approximately 4.5-month diet trial. Details of the diet trial, including exact diet composition, have been reported previously (14). Sex distribution was 55% male and 45% female. Recorded clinical diagnoses included atopic dermatitis (*n* = 6), borderline atopic dermatitis (*n* = 1), food-induced atopic dermatitis (*n* = 1), and osteoarthritis (*n* = 2). The study also included three healthy control dogs, but for one individual, only serum and EDTA plasma was collected, and not lithium-heparin plasma. The dogs' diet prior to the diet trial baseline was reported as percentages of four different diet types: kibble, wet processed, raw, and homecooked. In analyses that considered the samples collected at the diet trial baseline, only dogs who ate ≥95% raw (*n* = 5) and ≥95% kibble (*n* = 5) were included in the analyses. In the serum–urine cohort, fasted serum and urine samples were collected from eight dogs, all Staffordshire bull terriers (75% male, 25% female). Diagnoses of atopic dermatitis were recorded for all 8 dogs. Diet type was recorded as raw (*n* = 4) or dry (*n* = 4).

### 2.2 Ethics, consent, and permissions

Owners provided informed consent prior to inclusion. The study protocol was approved by the Animal Experiment Board in Finland (ELLA) under permit ESAVI/3244/04.10.07/2013.

### 2.3 Samples

Blood was collected from the jugular vein using a closed collection system (Vacutainer^®^ Safety-Lok™ Blood collection sets, Becton Dickinson, Meylan, France) into Vacuette^®^ tubes (3 ml EDTA, 3 ml lithium heparin, and 6 mL plain serum). Serum tubes were kept at room temperature for 30 min to clot prior to centrifugation. Serum and plasma were separated by centrifugation at 2,100 × g for 15 min; plasma tubes (EDTA and lithium-heparin) were centrifuged immediately after the visit using the same protocol, the average time between blood being drawn to centrifugation was approximately 15 min. Following separation, serum and plasma were aliquoted into 100 μl volumes to minimize freeze–thaw cycles and stored at −80°C. Urine was collected into factory-clean specimen jars and stored at −80°C in 5 ml tubes. Urine was not centrifuged before freezing, but after thawing it was vortexed and subsequently aliquoted into 100 μl samples for metabolomics analysis. All samples were collected in the fasting state.

### 2.4 Metabolomics analyses

Sample preparation for UPLC–MS/MS and vendor-side raw data processing were carried out by Finnish Institute for Molecular Medicine (FIMM) personnel, who returned metabolite concentration tables together with assay-level comments on metabolite reliability. For extraction, 10 μl of labeled internal standard mixture was added to 100 μl of serum, EDTA plasma, lithium-heparin plasma, or urine. Metabolites were extracted using four volumes of extraction solvent (1:4, sample:solvent; 100% acetonitrile with 1% formic acid). Extracts were loaded onto an Ostro™ 96-well plate (Waters Corporation, Milford, MA, USA) and filtered under vacuum (Δ pressure 300–400 mbar) for 2.5 min, yielding clarified extracts collected into a 96-well plate. Plates were sealed and placed in the LC autosampler for injection.

Targeted analysis was performed on an ACQUITY UPLC–MS/MS system (Waters Corporation, Milford, MA, USA) with a 2.1 × 100 mm, 1.7 μm BEH amide HILIC column held at 45°C and an autosampler at 5°C. The total run time was 14.5 min (including 2.5 min equilibration) at 600 μl/min. The gradient began with 2.5 min at 100% mobile phase B (ACN/H_2_O 90/10 (v/v), 20 mM ammonium formate, pH 3), transitioned to 100% mobile phase A (ACN/H_2_O 50/50 (v/v), ammonium formate, pH 3) over 10 min, held at 100% A for 2 min, and re-equilibrated for 2.5 min. Injection volume was 5 μl. Needle and seal wash procedures were applied between injections (strong wash: methanol/isopropanol/ACN/H_2_O 25/25/25/25 with 0.5% formic acid; weak wash: methanol/isopropanol/ACN/H_2_O 25/25/25/25 with 0.5% ammonium hydroxide; seal wash: methanol/H_2_O 90/10), using partial-loop with needle overfill injections.

A Xevo^®^ TQ-S triple quadrupole mass spectrometer (Waters Corporation, Milford, MA, USA) was operated with electrospray ionization in both positive and negative polarities with 20 ms polarity switching. Capillary voltage was 0.6 kV in both polarities; source and desolvation temperatures were 120°C and 650°C. Nitrogen was used as desolvation gas (1,000 L/h) and argon as collision gas (0.15 ml/min). Metabolites were quantified in multiple reaction monitoring (MRM) mode with compound-specific optimization of declustering potentials and collision energies. Data were acquired in MassLynx 4.1 and processed in TargetLynx; quantification used labeled internal standards and external calibration curves.

A full description of the targeted metabolomics method has been reported previously (18). The assay was performed using the laboratory's targeted Waters UPLC–MS/MS workflow (Waters Corporation, Milford, MA, USA), with the targeted metabolite panel and isotopic quantification framework based on the Biocrates AbsoluteIDQ p180 kit metabolite standard set (Biocrates Life Sciences AG, Innsbruck, Austria). In the biofluid workflow used here, 10 μl of labeled internal-standard mixture was added to 100 μl of biofluid, followed by protein precipitation with 400 μl of 1% formic acid in acetonitrile and LC–MS/MS analysis using external calibration curves and labeled internal-standard correction (18). In the prior validation study, this workflow was validated according to European Medicines Agency bioanalytical guidance for selectivity, specificity, linearity, accuracy, precision, extraction recovery, matrix effect, and stability, and the general biofluid protocol was described for blood, plasma, serum, cerebrospinal fluid, and urine (18). However, explicit biofluid validation experiments were performed in human serum rather than in canine serum, EDTA plasma, lithium-heparin plasma, or urine. The present study should therefore be regarded as a pilot application of this established workflow to canine biofluids rather than a *de novo* analytical validation in canine matrices. In the validated workflow, calibration was based on aqueous 11-point external calibration curves using analyte-to-labeled-standard peak-area ratios. Matrix effects were assessed using post-extraction spiked quality-control samples vs. aqueous spiked samples. Correction relied on 12 labeled internal standards representing chemically similar classes, with process-efficiency correction for analytes lacking a dedicated internal standard (18).

### 2.5 Statistical analysis

All preprocessing, statistical analyses, and figures were generated in R (v4.3 or later) using tidyverse-style data handling and custom scripts. Two matched-sample datasets were analyzed: (1) serum, EDTA plasma, and lithium-heparin plasma collected simultaneously from the same dogs (*n* = 11), and (2) serum and urine collected simultaneously (*n* = 8). All samples were fasting. Generative AI tools from OpenAI (ChatGPT, GPT-5.3 Thinking and GPT 5.4 Thinking, and GPT-5.3-Codex) were used to support editorial revision of the manuscript and limited code refactoring/debugging; all resulting text, code, analyses, and references were reviewed and verified by the authors.

### 2.6 Data preprocessing

Metabolite concentration tables and metadata were imported into R and harmonized across matrices by intersecting metabolite identifiers and aligning matched samples within each dog. Concentrations were transformed to log_10_ concentration. Missingness was assessed at two levels. First, metabolite-level missingness was quantified within each matrix (biofluid × anticoagulant) as the percentage of samples with missing concentration values. Metabolites were retained for analysis if less than 20% were missing in the relevant matrix, consistent with commonly used missingness thresholds in metabolomics preprocessing (19–21). For cross-matrix comparisons, metabolites were additionally required to satisfy this criterion in both matrices of a given pair (intersection set). Second, sample-level missingness was quantified within each matrix as the percentage of metabolites missing in that sample (on the log_10_ scale). Samples with >20% missing metabolite values within a given biofluid matrix were excluded from analyses involving that matrix, while retaining the same dog's measurements in other matrices that met the sample-level criterion (e.g., if a dog had 16% missingness in serum but 40% in lithium-heparin plasma, the lithium-heparin sample was excluded and serum retained). For multivariate analyses only, metabolites were further required to be detected in at least 80% of samples within the relevant matrix subset, and any remaining missing values were imputed using the within-matrix metabolite median. For multivariate diet analyses involving urine, urinary metabolite concentrations were normalized to the corresponding sample-specific urine creatinine concentration before log_10_ transformation; creatinine itself was not normalized.

Laboratory personnel comments, including observations that individual measurements were below the lower limit of quantification or above the upper limit of quantification, were reviewed but were not used as automatic exclusion criteria in the metabolite-level comparison analyses. Instead, filtering was based on predefined metabolite- and sample-level missingness thresholds, while assay-level reliability comments were retained as annotations, because the aim was to evaluate empirical cross-matrix agreement under the full analytical workflow. Laboratory comment sheets from both analytical time points, including assay-level annotations such as chromatographic comments and values reported below the lower or above the upper limit of quantification, are provided as supplementary data for transparency (Supplementary File 1).

### 2.7 Multivariate biosample comparison

Two complementary principal component analysis (PCA)-based multivariate analyses were performed. First, PCA was used to compare the overall metabolite profiles of the different biofluid sample types, thereby visualizing global matrix-driven differences between serum, EDTA plasma, lithium-heparin plasma, and, where applicable, urine. Second, PCA was performed within each biofluid matrix to evaluate how well dogs could be separated according to diet and, in exploratory analyses, health status within that matrix, thereby allowing comparison of which biofluids most clearly reflect diet- and health-associated variation in the metabolite profiles. Health-status PCA was restricted to serum and EDTA plasma because the number of healthy control dogs available for lithium-heparin plasma (*n* = 2) was insufficient for meaningful interpretation. In both settings, PCA was based on autoscaled metabolite profiles, and group dispersion was visualized with confidence ellipses derived from the covariance of the PCA scores. For the diet- and health-group analyses, group separation was further tested using permutational multivariate analysis of variance (PERMANOVA) via vegan::adonis2 on Euclidean distance matrices computed from the transformed and scaled data, using 9,999 permutations (22). We report the PERMANOVA pseudo-R^2^ from adonis2, representing the proportion of distance-matrix variance attributable to the predictor, together with the permutation *p*-value. In the serum–urine diet PCA/PERMANOVA analyses, urine values were creatinine-normalized as described above, whereas metabolite-level serum–urine comparability analyses were performed on unadjusted concentrations.

### 2.8 Metabolite-level cross-matrix agreement, correlation, and surrogate labels

Cross-matrix comparability was evaluated metabolite-by-metabolite using paired samples from the same dog. In dataset 1, matrix pairs comprised serum–EDTA plasma, serum–lithium-heparin plasma, and EDTA–lithium-heparin plasma. In dataset 2, the matrix pair comprised serum–urine.

Correlation was quantified using Pearson correlation coefficients. The number of complete paired observations per metabolite was recorded, and correlation statistics were only computed when at least three paired observations were available. For both dataset 1 and 2, Pearson correlation *p*-values were derived from the correlation t-statistic and adjusted across metabolites within each matrix pair using the Benjamini–Hochberg false discovery rate (BH-FDR).

Systematic matrix bias, i.e., systematic concentration differences between matrices, was assessed on the log_10_ scale using paired differences (right – left). For each metabolite, the mean paired log_10_ difference was calculated and then back-transformed to a fold change (10 raised to the mean paired log_10_ difference), with direction defined by the matrix-pair specification. Statistical evidence for bias was evaluated using a paired *t*-test on the paired differences and BH-FDR adjustment across metabolites within each matrix pair.

For the summary table of metabolite-level correlations with 95% confidence intervals, Pearson correlation coefficients (r) were extracted from the four pairwise biofluid comparison tables (serum vs. EDTA plasma, serum vs. lithium-heparin plasma, lithium-heparin vs. EDTA plasma, and serum vs. urine). Correlations were calculated from complete paired observations within each matrix pair after application of the predefined missingness filters, namely exclusion of samples with >20% missing metabolite values within a matrix and inclusion of metabolites with ≤ 20% missing values within the relevant matrix. Only metabolites present in all four pairwise comparison tables were retained for the final summary table.

Ninety-five percent confidence intervals for Pearson's r were then calculated using Fisher's z transformation. Confidence intervals were reported when n ≥ 4. If fewer paired observations were available or r was missing, only the correlation coefficient was reported or the cell was left blank, as appropriate.

To facilitate interpretation and downstream presentation, two rule-based labels were assigned for each metabolite and matrix pair. First, surrogate quality summarized cross-matrix tracking based on Pearson correlation strength, BH-FDR significance, and the minimum number of complete paired observations required for classification. Metabolites were classified as excellent surrogate when *r* ≥ 0.90 and *q* < 0.05, good surrogate when 0.80 ≤ *r* < 0.90 and *q* < 0.05, and moderate surrogate when 0.60 ≤ *r* < 0.80. All remaining metabolites with sufficient paired observations were classified as poor surrogate. Metabolites with too few complete paired observations were classified separately as having insufficient data (fewer than four paired observations in dataset 1 and fewer than three in dataset 2). Second, biofluid choice combined surrogate quality with the magnitude of the systematic cross-matrix difference, expressed as the absolute mean paired log_10_ difference. Difference bands were defined as negligible ( ≤ log_10_(1.2), approximately within ±20%), moderate (> log10(1.2) to ≤ log_10_(2), approximately within 2-fold), and large (> log_10_(2), i.e., greater than 2-fold). Metabolites with excellent surrogate quality and negligible difference were considered highly interchangeable between matrices, whereas those with good surrogate quality and negligible difference were considered acceptable for use in either matrix. Metabolites with moderate surrogate quality and negligible difference were considered cautiously interchangeable. For metabolites with excellent or good surrogate quality and moderate differences, the paired-difference test was used to distinguish metabolites for which a systematic matrix shift remained supported after BH-FDR adjustment from those for which such a shift was not supported. Metabolites with moderate surrogate quality and more than negligible bias, as well as metabolites with excellent or good surrogate quality but large bias, were also classified as directionally comparable rather than directly interchangeable. Metabolites with poor surrogate quality were classified as unsuitable for cross-matrix comparison. The exported comparison tables also retained the raw and BH-FDR-adjusted *P*-values for the paired-difference test, together with the derived indicators used to summarize bias (Supplementary File 2). A summary of the biofluid-choice framework is provided in Table 1, and the surrogate-label criteria are summarized in Table S1.

**Table 1:** Summary of the biofluid-choice framework.

Label	Surrogate quality (cross-matrix tracking)	Systematic difference between matrices (from |Δlog_10_|)	Label rule summary	Interpretation
Very high correlation	Excellent surrogate	Negligible (≤ log_10_(1.2))	Excellent tracking with only small average difference between matrices	The two matrices can be treated as effectively interchangeable for this metabolite.
High correlation	Good surrogate	Negligible ( ≤ log_10_(1.2))	Good tracking with only small average difference between matrices	The two matrices are generally comparable for this metabolite, although with slightly less confidence than the category above.
High correlation but requires calibration	Excellent surrogate or good surrogate	Moderate (> log_10_(1.2) to ≤ log_10_(2))	Strong tracking, but one matrix gives systematically higher or lower values	The metabolite tracks well across matrices, but raw concentrations should not be pooled without accounting for matrix-specific differences.
Moderate correlation	Moderate surrogate	Negligible ( ≤ log_10_(1.2))	Moderate tracking with only small average difference between matrices	The metabolite may be compared across matrices with caution, but cross-matrix agreement is less robust.
Directionally comparable	Moderate surrogate with non-negligible difference, **or** excellent/good surrogate with large difference	Non-negligible (> log_10_(1.2))	Values tend to move in the same direction across matrices, but absolute concentrations are not directly comparable	The metabolite may still provide directional information across matrices, but matrix-specific values should not be interpreted as interchangeable.
Avoid comparing	Poor surrogate	Any	Poor cross-matrix tracking	The metabolite should not be compared across matrices.

Surrogate quality was based on Pearson correlation strength and BH-adjusted significance: excellent surrogate, r ≥ 0.90 and q < 0.05; good surrogate, 0.80 ≤ r < 0.90 and q < 0.05; moderate surrogate, 0.60 ≤ r < 0.80; and poor surrogate, r < 0.60. Systematic difference between matrices was quantified as the absolute mean paired log_10_ difference (|Δlog_10_|). Negligible difference was defined as ≤ log_10_(1.2) (approximately within ±20%), moderate difference as > log_10_(1.2) to ≤ log_10_(2), and large difference as > log_10_(2) (greater than twofold). Bias significance metrics (p_bias, q_bias) were retained as annotations but were not used as primary gating criteria for category assignment.

## 3 Results

**Figure 1:**
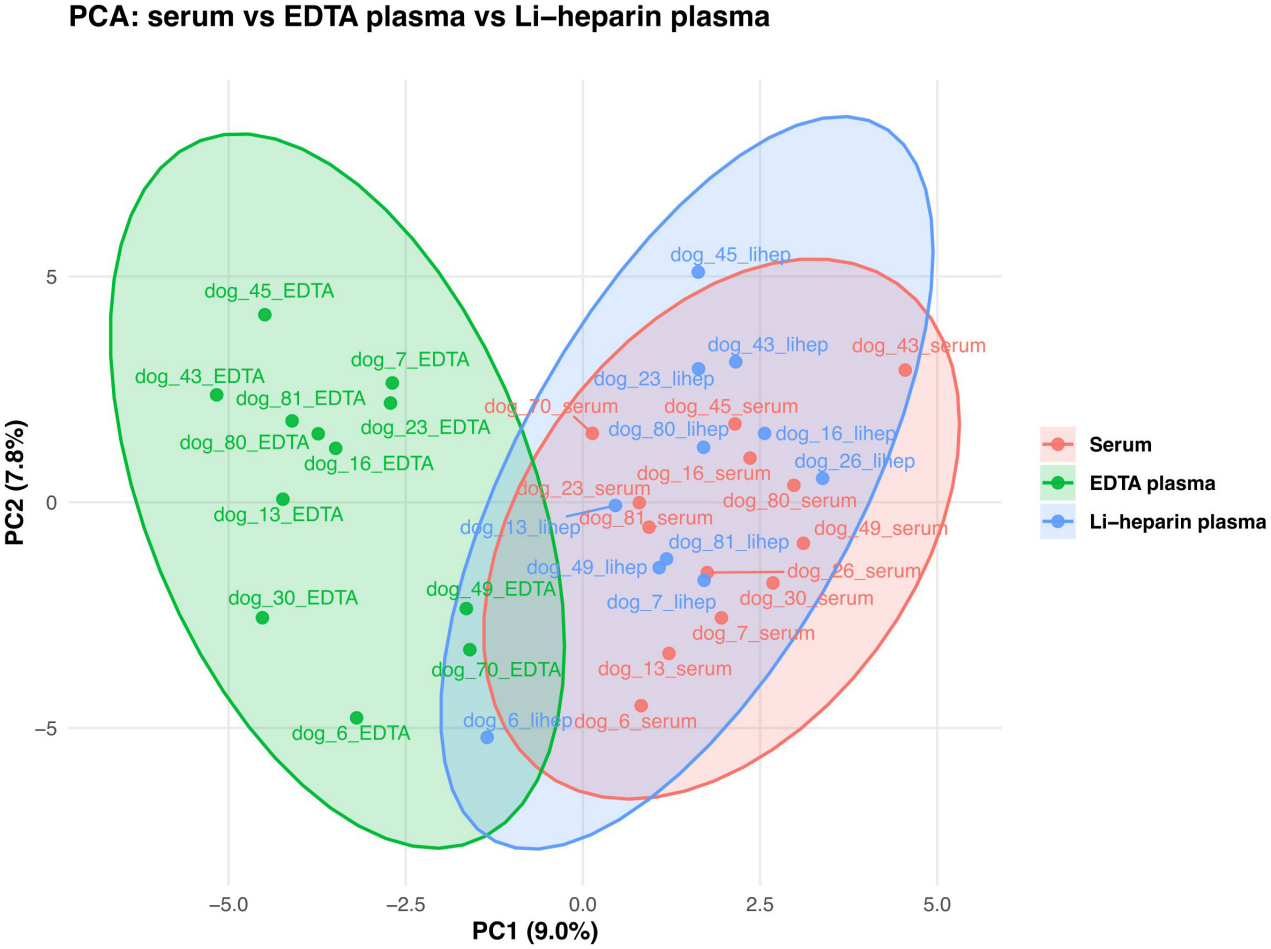
Principal component analysis (PCA) of metabolite profiles from the blood-matrix cohort showed clear separation of EDTA plasma from both serum and lithium-heparin plasma. In contrast, the 95% confidence ellipses, derived from the covariance of the PCA scores, for serum and lithium-heparin plasma overlapped extensively, indicating that these two matrices were globally more similar to each other than either was to EDTA plasma.

After applying the predefined missingness filters (exclusion of metabolites with >20% missing values within a matrix and exclusion of samples with >20% missing metabolite values within a given biofluid), 88 (86.3%) metabolites remained for serum, 85 (83.3%) for EDTA plasma, and 88 (86.3%) for lithium-heparin plasma in the blood-matrix cohort. Principal component analysis of the blood-based biofluids showed clear separation of EDTA plasma from both serum and lithium-heparin plasma, whereas serum and lithium-heparin plasma clustered closely and showed substantial overlap in the PCA score space (Figure 1). This indicated that, at the global metabolite-profile level, lithium-heparin plasma more closely resembled serum than EDTA plasma.

To further visualize systematic differences between matrices, heatmaps were generated for the blood-based biofluids alone (serum, EDTA plasma, and lithium-heparin plasma; Figure S1) and for all four matrices including urine (Figure S2). The blood-only heatmap was included to facilitate visual comparison among the three blood-derived sample types, whereas the four-matrix heatmap illustrates that urine differed much more strongly from the blood-based biofluids than the blood-based biofluids differed from one another.

### 3.1 Cross-matrix agreement and comparability

**Figure 2:**
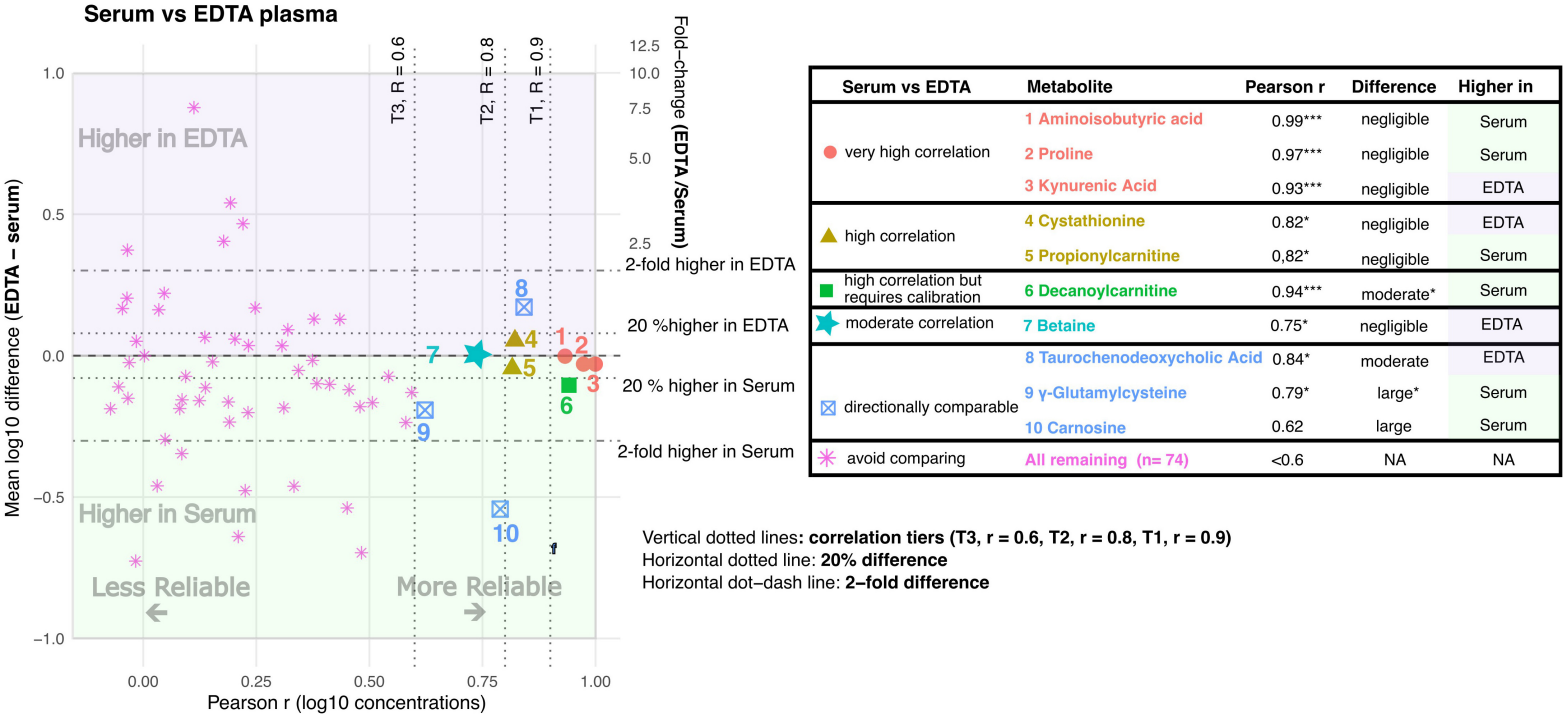
Cross-matrix agreement between EDTA plasma and serum. The scatterplot shows Pearson correlation (r, x-axis) against matrix difference (left y-axis, mean log_10_ difference EDTA – serum; right y-axis, fold change EDTA/serum). The table lists metabolites that were classified as comparable to varying degrees between EDTA plasma and serum. Asterisks indicate BH-FDR-adjusted significance levels for Pearson correlation and paired matrix difference: * adjusted *P* < 0.05, ** adjusted *P* < 0.01, and *** adjusted *P* < 0.001.

**Figure 3:**
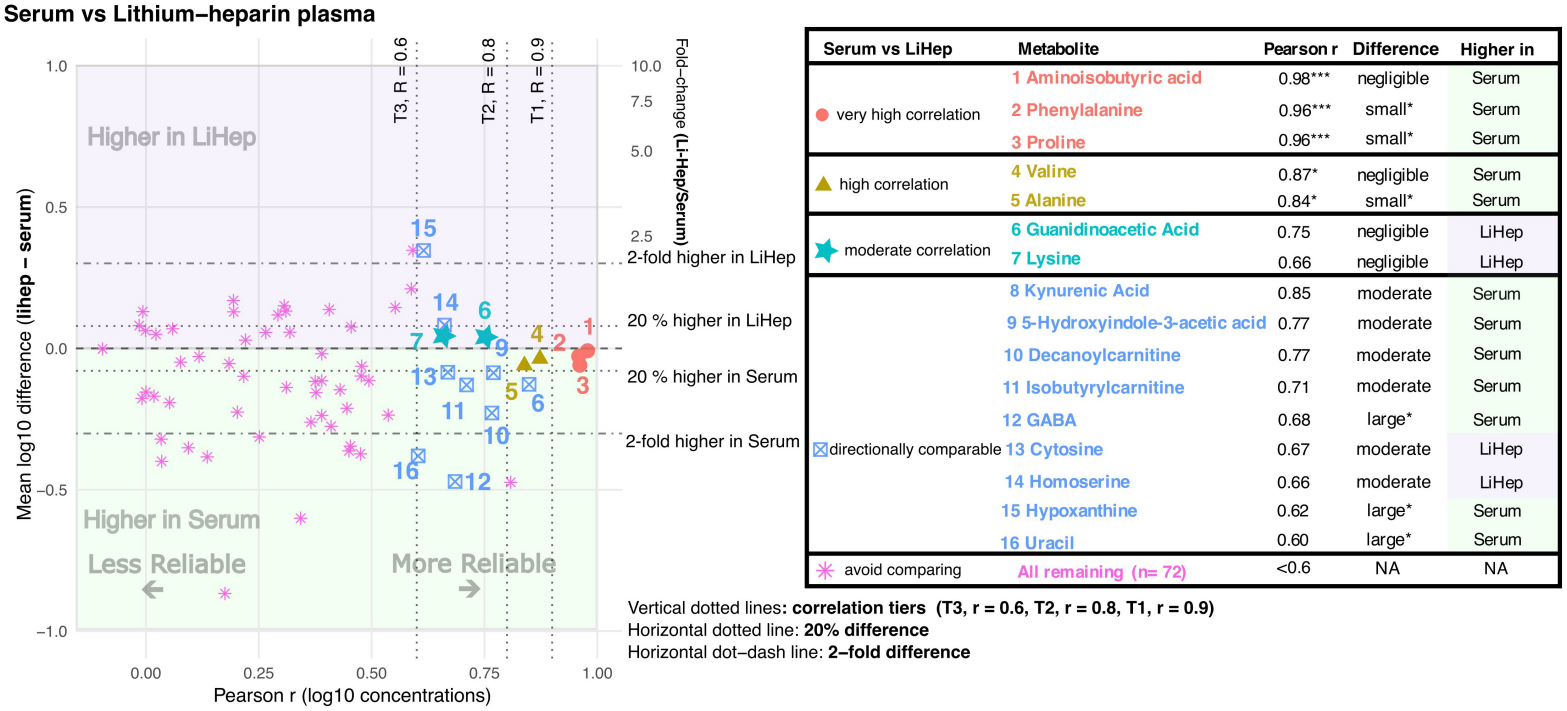
Cross-matrix agreement between lithium-heparin plasma and serum. The scatterplot shows Pearson correlation (r, x-axis) against matrix difference (left y-axis, mean log_10_ difference lithium-heparin – serum; right y-axis, fold change lithium-heparin/serum). The table lists metabolites that were classified as comparable to varying degrees between lithium-heparin plasma and serum. Asterisks indicate BH-FDR-adjusted significance levels for Pearson correlation and paired matrix difference: * adjusted *P* < 0.05, ** adjusted *P* < 0.01, and *** adjusted *P* < 0.001.

**Figure 4:**
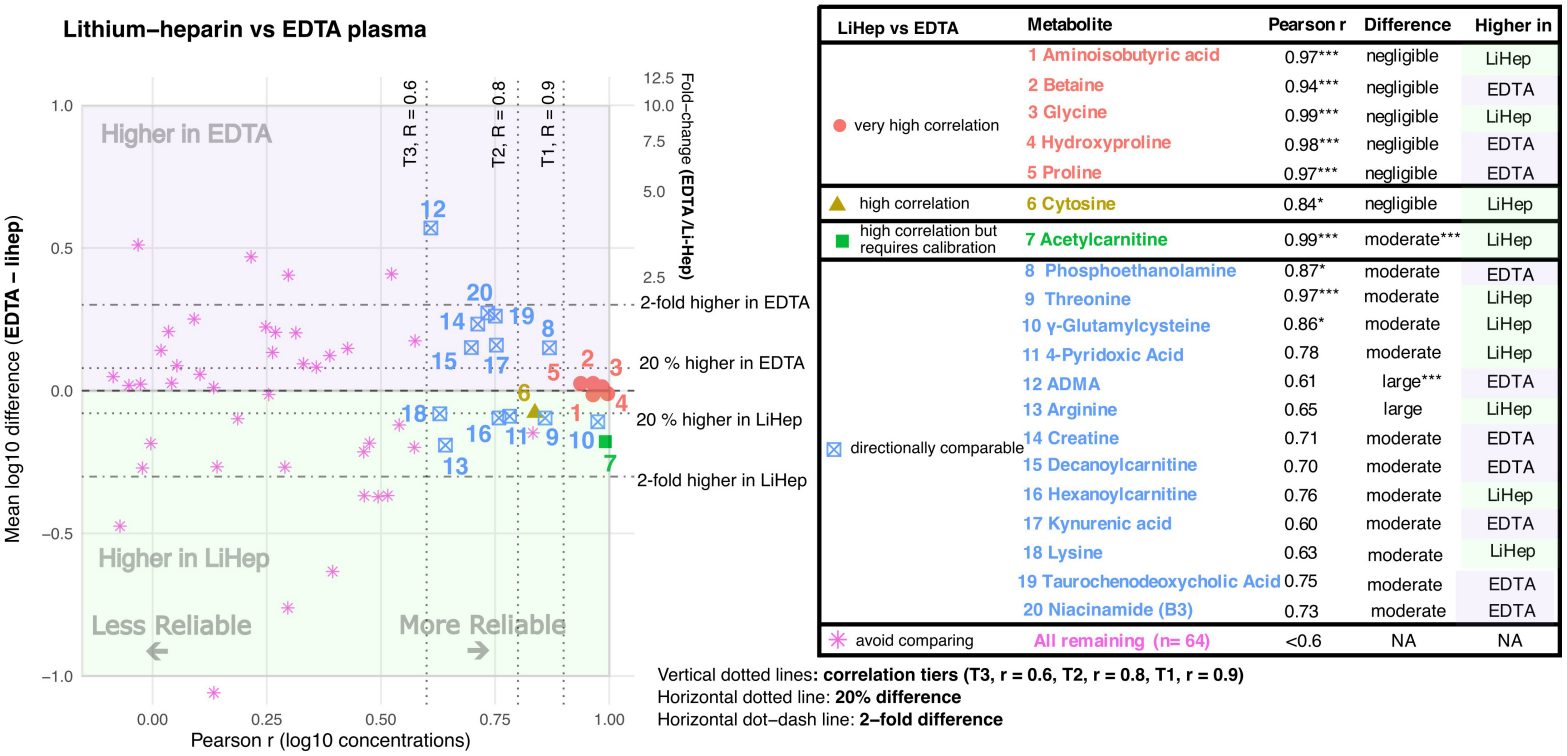
Cross-matrix agreement between EDTA plasma and lithium-heparin plasma. The scatterplot shows Pearson correlation (r, x-axis) against matrix difference (left y-axis, mean log_10_ difference EDTA – lithium-heparin; right y-axis, fold change EDTA/lithium-heparin). The table lists metabolites that were classified as comparable to varying degrees between EDTA plasma and lithium-heparin plasma. Asterisks indicate BH-FDR-adjusted significance levels for Pearson correlation and paired matrix difference: * adjusted *P* < 0.05, ** adjusted *P* < 0.01, and *** adjusted *P* < 0.001.

**Figure 5:**
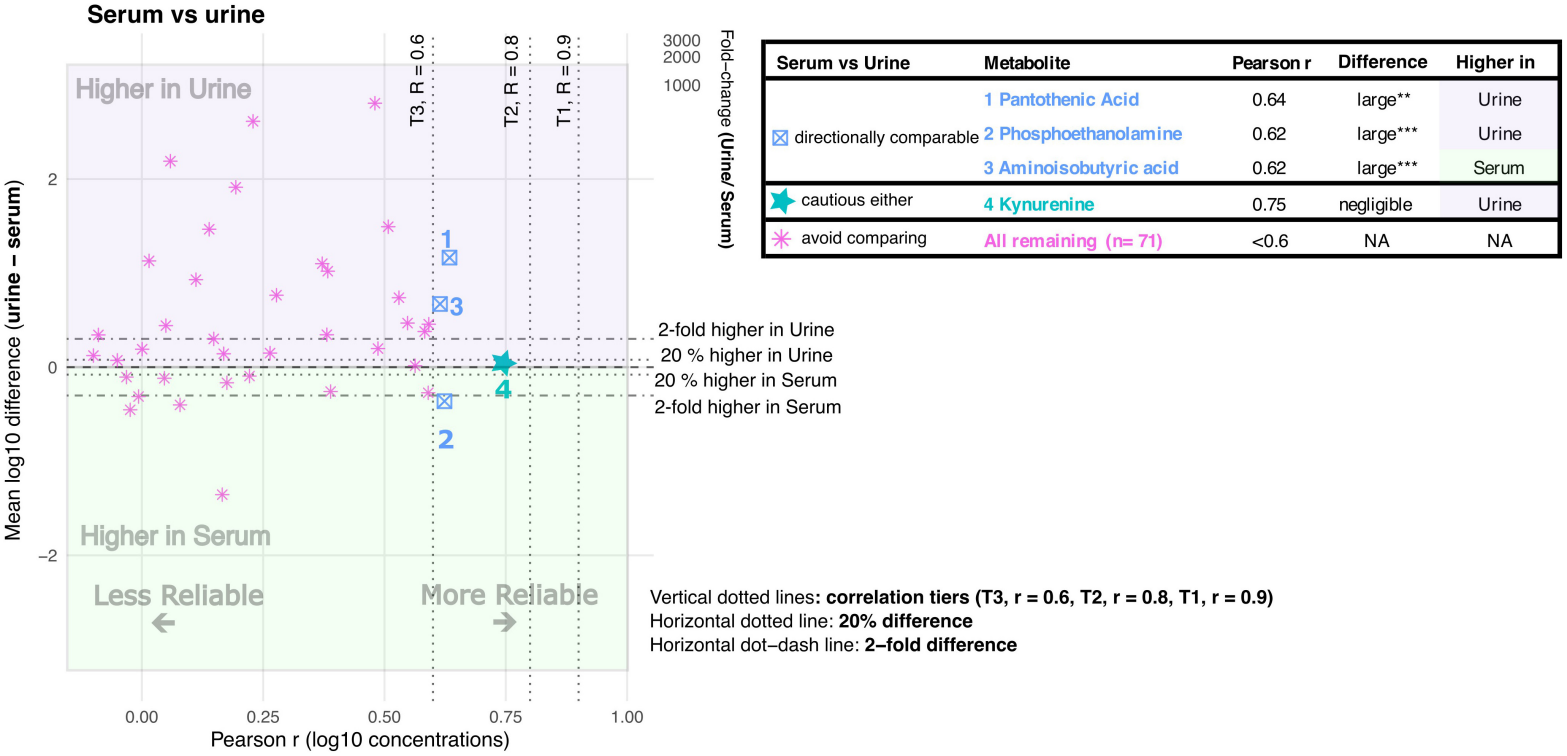
Cross-matrix agreement between urine and serum. The scatterplot shows Pearson correlation (r, x-axis) against matrix difference (left y-axis, mean log_10_ difference urine – serum; right y-axis, fold change urine/serum). The table lists metabolites that were classified as comparable to varying degrees between urine and serum. Asterisks indicate BH-FDR-adjusted significance levels for Pearson correlation and paired matrix difference: * adjusted *P* < 0.05, ** adjusted *P* < 0.01, and *** adjusted *P* < 0.001.

For each metabolite and matrix pair, Pearson correlations across paired dogs and the corresponding mean paired log_10_ concentration differences were calculated to assess cross-matrix agreement and systematic matrix-specific differences. Metabolites were then classified into comparability categories based on detection rate, correlation magnitude, and estimated matrix differences. The full set of metabolite comparisons across biofluids is presented in Table 2, and the distribution of comparable and non-comparable metabolites is further illustrated in Figures 2–5.

**Table 2:** Pearson correlation coefficients (*r*) and 95% Fisher z confidence intervals for metabolite concentrations across the four pairwise biofluid comparisons.

Metabolite Class	Metabolite	Pearson r (95%CI) for serum vs. EDTA	Pearson r (95%CI) for serum vs. LiHep	Pearson r (95%CI) for LiHep vs. EDTA	Pearson r (95%CI) for serum vs. urine
Acylcarnitines	Acetylcarnitine	0.19 (−0.43–0.69)	0.08 (−0.55–0.65)	**0.99 (0.97**–**1.00)**	−0.63 (−0.92–0.14)
	Decanoylcarnitine	**0.94 (0.80**–**0.98)**	**0.77 (0.31**–**0.94)**	**0.70 (0.12**–**0.92)**	−0.64 (−0.93–0.12)
	Hexanoylcarnitine	0.46 (−0.16–0.82)	0.54 (−0.09–0.86)	**0.76 (0.25**–**0.94)**	0.38 (−0.44–0.86)
	Isobutyrylcarnitine	0.09 (−0.51–0.63)	**0.71 (0.19**–**0.92)**	−0.02 (−0.64–0.62)	−0.53 (−0.90–0.28)
	Isovalerylcarnitine	0.31 (−0.32–0.75)	0.25 (−0.41–0.74)	0.36 (−0.35–0.81)	0.00 (−0.70–0.70)
	Octanoylcarnitine	0.54 (−0.05–0.85)	0.31 (−0.36–0.77)	0.46 (−0.24–0.85)	−0.51 (−0.89–0.31)
	Propionylcarnitine	**0.82 (0.46**–**0.95)**	0.02 (−0.59–0.61)	0.31 (−0.39–0.79)	−0.58 (−0.91–0.20)
Amino acids and derivatives	2–Aminodipic acid	0.58 (0.01–0.87)	−0.01 (−0.61–0.59)	0.19 (−0.50–0.73)	−0.33 (−0.84–0.49)
	ADMA	0.11 (−0.49–0.64)	0.59 (−0.01–0.88)	**0.61 (**–**0.03**–**0.90)**	0.38 (−0.44–0.86)
	Alanine	−0.68 (−0.90–0.17)	**0.84 (0.48**–**0.96)**	−0.52 (−0.87–0.17)	0.26 (−0.54–0.82)
	Aminoisobutyric acid	**0.99 (0.97**–**1.00)**	**0.98 (0.92**–**0.99)**	**0.97 (0.86**–**0.99)**	**0.62 (**–**0.16**–**0.92)**
	Arginine	−0.68 (−0.90–0.18)	−0.54 (−0.86–0.09)	**0.64 (0.02**–**0.91)**	−0.26 (−0.82–0.54)
	Asparagine	0.05 (−0.54–0.60)	−0.14 (−0.69–0.50)	−0.28 (−0.77–0.42)	0.55 (−0.26–0.90)
	Aspartate	0.20 (−0.42–0.70)	0.43 (−0.23–0.82)	0.03 (−0.61–0.65)	−0.44 (−0.87–0.39)
	Creatine	−0.41 (−0.80–0.21)	0.29 (−0.37–0.76)	**0.71 (0.15**–**0.93)**	−0.09 (−0.75–0.66)
	Creatinine	0.03 (−0.55–0.60)	0.19 (−0.46–0.71)	−0.03 (−0.65–0.61)	−0.60 (−0.92–0.17)
	Cystathionine	**0.82 (0.47**–**0.95)**	0.37 (−0.30–0.79)	0.25 (−0.45–0.76)	−0.10 (−0.75–0.65)
	Dimethyl glycine	0.34 (−0.29–0.77)	−0.27 (−0.75–0.39)	−0.55 (−0.88–0.12)	0.22 (−0.57–0.80)
	Glutamic acid	−0.40 (−0.79–0.22)	−0.41 (−0.81–0.26)	0.29 (−0.42–0.78)	−0.40 (−0.86–0.42)
	Glutamine	−0.13 (−0.66–0.48)	−0.10 (−0.66–0.53)	−0.19 (−0.73–0.50)	−0.11 (−0.76–0.64)
	Glycine	0.08 (−0.52–0.63)	0.37 (−0.29–0.80)	**0.99 (0.96**–**1.00)**	0.17 (−0.61–0.78)
	Guanidinoacetic acid	0.15 (−0.46–0.67)	**0.75 (0.28**–**0.93)**	−0.16 (−0.72–0.52)	−0.41 (−0.86–0.42)
	Histidine	−0.05 (−0.60–0.54)	−0.16 (−0.69–0.49)	−0.17 (−0.72–0.52)	0.05 (−0.68–0.73)
	Hydroxyproline	0.38 (−0.25–0.78)	0.19 (−0.46–0.71)	**0.98 (0.93**–**1.00)**	0.18 (−0.60–0.78)
	Isoleucine	−0.02 (−0.58–0.56)	0.39 (−0.27–0.80)	−0.03 (−0.65–0.61)	−0.32 (−0.84–0.50)
	Kynurenic acid	**0.93 (0.77**–**0.98)**	**0.85 (0.50**–**0.96)**	**0.75 (0.23**–**0.94)**	0.53 (−0.28–0.90)
	Kynurenine	0.51 (−0.09–0.84)	0.48 (−0.17–0.84)	0.13 (−0.54–0.70)	**0.75 (0.09**–**0.95)**
	Leucine	−0.32 (−0.75–0.32)	0.41 (−0.26–0.81)	−0.40 (−0.82–0.31)	−0.05 (−0.73–0.68)
	Lysine	0.37 (−0.25–0.78)	**0.66 (0.10**–**0.90)**	**0.63 (**–**0.00**–**0.90)**	−0.25 (−0.81–0.55)
	Methionine	0.25 (−0.38–0.72)	−0.11 (−0.66–0.53)	0.39 (−0.32–0.82)	−0.23 (−0.80–0.57)
	Phenylalanine	−0.07 (−0.62–0.52)	**0.96 (0.85**–**0.99)**	−0.00 (−0.63–0.63)	0.08 (−0.66–0.74)
	**Proline**	**0.97 (0.91**–**0.99)**	**0.96 (0.85**–**0.99)**	**0.97 (0.86**–**0.99)**	0.56 (−0.23–0.91)
	SDMA	−0.04 (−0.60–0.55)	−0.37 (−0.80–0.29)	0.26 (−0.44–0.77)	−0.27 (−0.82–0.54)
	Serine	0.48 (−0.13–0.83)	0.59 (−0.02–0.88)	0.46 (−0.23–0.85)	−0.30 (−0.83–0.51)
	Spermidine	−0.17 (−0.68–0.45)	0.45 (−0.20–0.83)	0.14 (−0.54–0.71)	0.17 (−0.61–0.78)
	Threonine	−0.12 (−0.65–0.49)	−0.12 (−0.67–0.52)	**0.97 (0.89**–**0.99)**	−0.14 (−0.77–0.63)
	Tryptophan	−0.24 (−0.71–0.39)	−0.28 (−0.75–0.38)	0.30 (−0.41–0.78)	−0.12 (−0.76–0.64)
	Tyrosine	−0.30 (−0.75–0.33)	−0.24 (−0.73–0.42)	0.09 (−0.57–0.68)	−0.16 (−0.78–0.62)
	Valine	−0.29 (−0.74–0.34)	**0.87 (0.57**–**0.97)**	−0.17 (−0.72–0.52)	−0.36 (−0.85–0.46)
Bile acids	Chenodeoxycholic acid	−0.51 (−0.84–0.09)	−0.29 (−0.76–0.38)	0.10 (−0.56–0.69)	−0.36 (−0.85–0.47)
	Glycocholic acid	0.41 (−0.25–0.81)	0.03 (−0.64–0.68)	0.57 (−0.22–0.91)	−0.22 (−0.80–0.57)
	Taurine	−0.04 (−0.60–0.55)	−0.42 (−0.81–0.25)	0.43 (−0.28–0.83)	0.58 (−0.21–0.91)
Carbohydrates	Sorbitol	−0.03 (−0.59–0.55)	0.06 (−0.56–0.64)	0.47 (−0.22–0.85)	0.59 (−0.19–0.91)
Choline metabolites/Others	Allantoin	0.43 (−0.19–0.81)	−0.21 (−0.72–0.44)	−0.22 (−0.75–0.48)	−0.37 (−0.85–0.45)
	Betaine	**0.75 (0.31**–**0.92)**	0.45 (−0.20–0.83)	**0.94 (0.75**–**0.99)**	−0.33 (−0.84–0.49)
	Carnitine	0.32 (−0.31–.76)	0.39 (−0.27–0.80)	−0.40 (−0.82–0.31)	0.49 (−0.33–0.89)
	Carnosine	**0.62 (0.08**–**0.88)**	−0.16 (−0.69–0.49)	0.02 (−0.62–0.64)	−0.01 (−0.71–0.70)
	Choline	0.19 (−0.43–0.69)	−0.00 (−0.60–0.60)	−0.24 (−0.75–0.46)	0.15 (−0.62–0.77)
Enzyme cofactors	4–Pyridoxic acid	−0.05 (−0.61–0.54)	0.02 (−0.59–0.61)	**0.78 (0.30**–**0.95)**	0.48 (−0.34–0.89)
	Glutathione (reduced)	0.22 (−0.40–0.71)	0.12 (−0.52–0.67)	0.52 (−0.16–0.87)	0.01 (−0.75–0.76)
	Niacinamide (B3)	0.18 (−0.44–0.68)	−0.02 (−0.61–0.59)	**0.73 (0.19**–**0.93)**	0.11 (−0.64–0.76)
	Pantothenic acid	−0.22 (−0.70–0.41)	0.20 (−0.45–0.72)	−0.40 (−0.82–0.31)	0.64 (−0.12–0.93)
Ethanolamines	Phosphoethanolamine	0.23 (−0.39–0.71)	0.03 (−0.64–0.68)	**0.87 (0.42**–**0.98)**	0.62 (−0.25–0.94)
Neurotransmitter metabolic intermediates	GABA	−0.39 (−0.79–0.24)	**0.68 (0.14**–**0.91)**	−0.38 (−0.81–0.33)	0.37 (−0.53–0.88)
	Normetanephrine	0.38 (−0.24–0.78)	0.05 (−0.57–0.63)	−0.05 (−0.66–0.60)	0.23 (−0.57–0.80)
Nucleobases	Adenine	0.48 (−0.21–0.85)	0.38 (−0.29–0.80)	0.39 (−0.43–0.86)	0.19 (−0.65–0.83)
	Cytosine	0.59 (0.03–0.87)	**0.67 (0.11**–**0.91)**	**0.84 (0.45**–**0.96)**	0.06 (−0.67–0.73)
	Hypoxanthine	−0.23 (−0.71–0.39)	**0.62 (0.02**–**0.89)**	0.30 (−0.41–0.78)	−0.03 (−0.72–0.69)
	Orotic acid	0.14 (−0.47–0.66)	0.49 (−0.15–0.84)	0.04 (−0.60–0.65)	0.14 (−0.63–0.77)
	Uracil	−0.17 (−0.68–0.45)	**0.60 (0.01**–**0.88)**	0.27 (−0.43–0.77)	0.05 (−0.68–0.73)
	Xanthine	−0.02 (−0.58–0.56)	0.00 (−0.60–0.60)	−0.20 (−0.74–0.49)	−0.16 (−0.78–0.61)
	2-deoxycytidine	0.09 (−0.51–0.63)	0.14 (−0.51–0.68)	0.05 (−0.60–0.66)	0.59 (−0.20–0.91)
	Adenosine	−0.24 (−0.71–0.39)	0.55 (−0.07–0.87)	−0.17 (−0.72–0.51)	−0.20 (−0.79–0.59)
	Inosine	−0.21 (−0.70–0.41)	0.34 (−0.32–0.78)	0.13 (−0.54–0.70)	−0.18 (−0.78–0.60)
	AMP	−0.57 (−0.86–0.00)	0.39 (−0.27–0.80)	−0.58 (−0.88–0.08)	−0.41 (−0.86–0.42)
Organic compounds	1-methylhistamine	0.03 (−0.55–0.59)	0.09 (−0.54–0.66)	−0.67 (−0.91–0.06)	0.51 (−0.31–0.89)
	Glucoronate	0.21 (−0.41–0.70)	0.48 (−0.17–0.84)	−0.07 (−0.67–0.58)	−0.44 (−0.87–0.38)
	Hippuric acid	0.23 (−0.40–0.71)	0.48 (−0.17–0.84)	−0.11 (−0.69–0.56)	−0.34 (−0.84–0.48)
	Trimethylamine-N-Oxide	0.31 (−0.32–0.75)	0.32 (−0.35–0.77)	0.25 (−0.45–0.76)	−0.14 (−0.77–0.63)
TCA cycle intermediates	Succinate	0.33 (−0.30–0.76)	0.18 (−0.47–0.71)	−0.25 (−0.76–0.45)	−0.34 (−0.84–0.48)
Urea cycle intermediates	Citrulline	−0.03 (−0.60–0.55)	−0.39 (−0.80–0.28)	−0.09 (−0.68–0.57)	−0.02 (−0.72–0.69)
	Ornithine	0.05 (−0.54–0.61)	−0.17 (−0.70–0.48)	−0.23 (−0.75–0.46)	−0.36 (−0.85–0.46)

Green highlighting denotes metabolite pairs with very high cross-matrix correlation (*r* > 0.90) and a lower 95% confidence bound >0.60. Orange highlighting denotes metabolite pairs with high observed correlation (*r* > 0.80) but a lower 95% confidence bound < 0.60.

Bold values indicate Pearson correlation coefficients of *r* > 0.60, corresponding to at least moderate cross-matrix correlation. Boldface refers only to the correlation threshold and does not by itself indicate direct interchangeability between biofluid matrices.

CI, Confidence interval, LiHep, Lithium-heparin plasma.

Of the 84 serum–EDTA plasma metabolite comparisons (Figure 2), 10 (11.9%) were comparable to varying degrees, whereas 74 (88.1%) were classified as not suitable for cross-matrix comparison. Aminoisobutyric acid, proline, and kynurenic acid showed the best interchangeability between the two blood sample types. Cystathionine and propionylcarnitine also showed high correlation (Pearson *r* > 0.8, *q* < 0.05), and for all five metabolites the concentration differences between EDTA plasma and serum appeared negligible. Decanoylcarnitine showed very high correlation but required calibration, as concentrations were consistently and significantly higher in serum than in EDTA plasma. Betaine showed negligible concentration differences between serum and EDTA plasma, but only moderate correlation (Pearson *r* = 0.75). Taurochenodeoxycholic acid concentrations were higher in EDTA plasma, whereas γ-glutamylcysteine and carnosine concentrations were higher in serum. These three metabolites showed varying degrees of correlation, but the moderate to large and unreliable concentration differences between EDTA plasma and serum indicate that they are directionally comparable rather than interchangeable.

Of the 88 metabolite comparisons between serum and lithium-heparin plasma (Figure 3), 16 (18.2%) were comparable to varying degrees, whereas 72 (81.8%) were classified as not suitable for cross-matrix comparison. Aminoisobutyric acid, proline, and alanine showed the best interchangeability between these two matrices. Valine and alanine also showed high correlation between serum and lithium-heparin plasma. Small (< 20%) but reliable concentration shifts were detected for phenylalanine, proline, and alanine, with higher concentrations in serum, whereas the concentration differences of aminoisobutyric acid and valine were negligible. Guanidinoacetic acid and lysine showed negligible concentration differences between the two biofluids, but only moderate correlation (Pearson *r* = 0.75 and *r* = 0.66, respectively). Finally, 5-hydroxyindole-3-acetic acid, cytosine, decanoylcarnitine, GABA, isobutyrylcarnitine, kynurenic acid, and uracil showed varying degrees of correlation together with moderate to large concentration differences toward serum, whereas homoserine and hypoxanthine showed a moderate shift toward lithium-heparin plasma. These findings indicate that for these metabolites serum and lithium-heparin plasma are directionally comparable, but not interchangeable.

Of the 84 metabolite comparisons between lithium-heparin and EDTA plasma (Figure 4), 20 (23.8%) were comparable to varying degrees, whereas 64 (76.2%) were classified as not suitable for cross-matrix comparison. Aminoisobutyric acid, betaine, glycine, hydroxyproline, and proline showed very high correlation, and cytosine showed high correlation, all with negligible concentration differences (< 20%) between the two plasma types. Acetylcarnitine showed very high correlation between lithium-heparin and EDTA plasma (Pearson *r* = 0.99). Phosphoethanolamine, ADMA, creatine, kynurenic acid, taurochenodeoxycholic acid, and niacinamide concentrations were all higher in EDTA plasma, whereas threonine, γ-glutamylcysteine, 4-pyridoxic acid, arginine, decanoylcarnitine, hexanoylcarnitine, and lysine concentrations were higher in lithium-heparin plasma. These metabolites showed varying degrees of correlation together with moderate to large concentration differences, indicating that their concentrations in EDTA plasma and lithium-heparin plasma are directionally comparable, but not interchangeable.

Of the 75 metabolite comparisons between urine and serum (Figure 5), four (5.3%) were comparable to varying degrees, whereas 71 (94.7%) were classified as not suitable for cross-matrix comparison. Pantothenic acid and phosphoethanolamine both showed reliably higher concentrations (≥2-fold) in urine than in serum, but only modest correlation (*r* = 0.64 and *r* = 0.62, respectively). Aminoisobutyric acid showed a reliably higher concentration (≥2-fold) in serum than in urine, but likewise only modest correlation (Pearson *r* = 0.62). These results indicate that these metabolites are directionally comparable between serum and urine, but not interchangeable. Kynurenine showed negligible concentration differences between serum and urine, but only moderate correlation (Pearson *r* = 0.75), indicating that although serum and urine concentrations may be comparable for this metabolite, such comparisons should be interpreted cautiously.

### 3.2 Diet- and health-associated separation differed across biosample types

**Figure 6:**
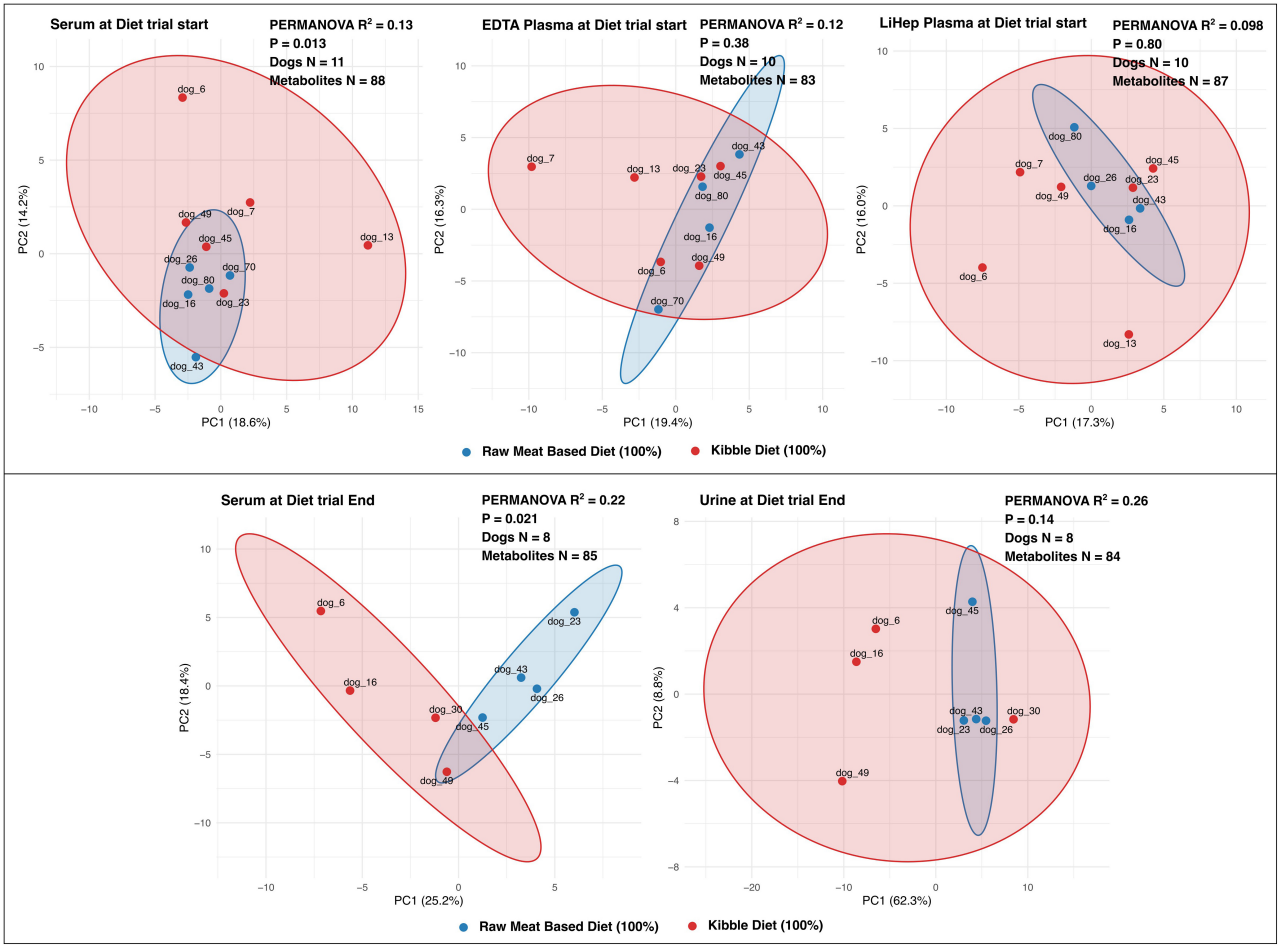
Principal component analysis (PCA) plots showing diet-associated separation of metabolite profiles across biofluids. The upper row shows the baseline blood-matrix cohort (serum, EDTA plasma, and lithium-heparin plasma), and the lower row shows the end-of-trial serum–urine cohort. Points represent individual dogs, and 95% confidence ellipses are derived from the covariance of the PCA scores. Panel annotations report PERMANOVA pseudo-R^2^, permutation *p*-value, number of dogs, and number of metabolites included.

Because dietary composition was recorded at the individual level rather than representing a matrix-specific attribute, PCA was used to examine how diet-associated variation was reflected across biofluids (Figure 6). At the baseline timepoint of the diet trial, serum showed the clearest diet-associated separation among the blood-based matrices and was the only matrix with statistically supported separation by PERMANOVA (pseudo-*R*^2^ = 0.13, *p* = 0.013), whereas EDTA plasma (pseudo-*R*^2^ = 0.12, *p* = 0.38) and lithium-heparin plasma (pseudo-*R*^2^ = 0.098, *p* = 0.80) showed weaker and non-significant separation. At the end of the diet trial, after approximately 4.5 months of controlled feeding, serum again showed statistically supported diet-associated separation (pseudo-*R*^2^ = 0.22, *p* = 0.021). Urine showed a numerically similar, or slightly larger, pseudo-*R*^2^ (0.26) but this did not reach statistical significance (*p* = 0.14), indicating suggestive but less robust diet-associated separation than in serum.

Health-associated separation between healthy control dogs and dogs with atopic dermatitis was weak and should be interpreted as exploratory because of the limited and imbalanced group sizes (Figure S3). In the baseline blood-matrix cohort, serum showed a PERMANOVA pseudo-*R*^2^ of 0.085 (*p* = 0.89) and EDTA plasma a pseudo-*R*^2^ of 0.092 (*p* = 0.83), indicating no statistically supported separation by health status. Health-group analysis in lithium-heparin plasma was not interpretable because too few healthy control samples were available.

## 4 Discussion

### 4.1 Main findings

To our knowledge, this is the first study to compare targeted small polar metabolite profiles across matched canine serum, EDTA plasma, lithium-heparin plasma, and urine samples. Three main findings emerged. First, metabolite comparability was strongly matrix- and metabolite-dependent. Second, among the blood-based matrices, serum and lithium-heparin plasma were globally more similar to each other than either was to EDTA plasma. Third, serum most clearly reflected diet-associated separation, whereas health-associated separation was weak and exploratory.

These findings show that biospecimen choice is not a minor pre-analytical detail, but a determinant of whether metabolite concentrations can be interpreted interchangeably across matrices. They also emphasize that strong cross-matrix correlation does not necessarily imply equivalence of absolute concentrations, because systematic matrix-dependent shifts were common even among metabolites with good or excellent correlation.

### 4.2 Cross-matrix comparability among blood-based biofluids

Among the blood-based matrices, serum and lithium-heparin plasma were globally more similar to each other than either was to EDTA plasma. This pattern is consistent with previous work showing that serum and plasma should not be treated as interchangeable categories, and that the choice of anticoagulant can materially influence metabolite measurements (1). In practical terms, the present results suggest that EDTA plasma differs more substantially from serum than lithium-heparin plasma does for this targeted panel of small polar metabolites.

At the metabolite level, only a minority of analytes were directly interchangeable between matrices. Most metabolites were classified as not suitable for direct cross-matrix comparison, and even within the subset that tracked well, matrix-specific concentration shifts were common. Thus, a substantial number of metabolites were best regarded as directionally comparable rather than directly interchangeable. For clinical and translational interpretation, this means that a metabolite may still be useful for identifying relative differences between dogs across matrices, while remaining unsuitable for pooling, reference interval transfer, or direct substitution without adjustment.

The greater divergence of EDTA plasma from serum is biologically plausible. During serum preparation, the clotting period before centrifugation permits ongoing cellular and platelet metabolism, which can alter the abundance of some small polar metabolites relative to plasma. By contrast, EDTA suppresses clotting through chelation of calcium and magnesium, thereby also affecting some metal-dependent enzymatic processes and potentially influencing analytes beyond the simple prevention of coagulation (1, 3). Lithium-heparin acts through the antithrombin pathway without strong chelation and may therefore preserve a metabolic profile that is globally closer to serum for some analytes in this targeted panel. These mechanisms provide a plausible pre-analytical explanation for why serum and lithium-heparin plasma were more similar to each other overall than either was to EDTA plasma, while still emphasizing that matrix effects remained strongly metabolite-specific.

The present findings therefore support the view that “plasma” should not be treated as a single generic biospecimen category in metabolomics. Instead, EDTA plasma and lithium-heparin plasma should be regarded as analytically distinct matrices whose comparability with serum must be established empirically. Although no metabolite class showed uniformly good interchangeability across matrices, some broad class-level tendencies were apparent.

The metabolites showing the most favorable agreement across the blood-based matrices were the amino acids aminoisobutyric acid, proline, alanine, glycine, and hydroxyproline, the amino acid derivative kynurenic acid, and betaine. In contrast, a subset of other small polar metabolites including several bile acid derivatives, nucleobase- or nucleoside-related metabolites more often showed matrix-dependent concentration shifts despite sometimes retaining moderate to strong cross-matrix correlation. The acylcarnitines acetylcarnitine, isobutyrylcarnitine, hexanoylcarnitine, and decanoylcarnitine tracked well across matrices, but systematic differences were common, such that they were more often directionally comparable than directly interchangeable. Overall, these findings suggest that matrix effects were partly structured by metabolite chemistry but were metabolite-specific. Metabolite class alone was not a reliable basis for assuming cross-matrix comparability.

### 4.3 Urine as a distinct biospecimen

Serum and urine showed the lowest metabolite-level comparability, both globally and for individual analytes. This was expected. Unlike blood which is kept in tight homeostasis, urine is an excretory matrix and its composition is strongly influenced by hydration state, renal handling, and excretion dynamics (9).

These results argue against using urine as a direct surrogate for blood-based matrices in targeted profiling of small polar metabolites. Instead, urine should be considered a biologically distinct and complementary matrix. In some contexts, particularly for excreted or diet-responsive metabolites, urine may provide additional information that is not evident in serum or plasma. However, it should not be interpreted as directly interchangeable with blood-based matrices for this assay.

### 4.4 Diet- and health-associated variation across biofluids

A further aim of this study was to examine how well different biofluids captured biologically meaningful between-dog variation. Serum most clearly reflected diet-associated separation. At baseline, serum was the only blood-based matrix showing statistically supported diet-group separation, and at the end of the dietary intervention serum again showed significant separation. Urine showed a numerically similar, or slightly larger, pseudo-*R*^2^ at the end timepoint, but the separation was not statistically supported. Because the serum-urine cohort was sampled at a later, more tightly controlled stage of the diet trial, direct ranking of urine against the plasma matrices is not justified. Nevertheless, the present results support serum as a robust choice for detecting diet-associated differences with this targeted assay.

These findings are consistent with our previous findings (11), which showed that diet-associated variation was more readily detectable than disease-associated variation in serum and urine using the same targeted metabolite panel. Taken together, the earlier and present results suggest that habitual or controlled dietary differences exert a stronger influence on these small polar metabolite profiles than the atopic dermatitis phenotype captured here. Health-associated separation between dogs with atopic dermatitis and healthy controls was weak and should be interpreted cautiously. This likely reflects a combination of the small and imbalanced health groups, the resulting limited statistical power, and that the present targeted panel appears better suited to detecting diet-related metabolic variation than inflammatory or disease-related variation. The lack of clear multivariate separation should therefore not be interpreted as evidence that health status had no metabolic effects, but rather that this study was not sufficiently powered or analytically optimized to resolve them. Accordingly, the present data do not support strong conclusions regarding an optimal biofluid for distinguishing atopic from healthy dogs with this assay.

### 4.5 Strengths and limitations

This study has several strengths. First, the matched-sample design reduced between-dog biological variability when comparing biospecimens. Second, all matrices were analyzed using a common targeted platform, allowing direct methodological comparison. Third, comparability was evaluated using both correlation and systematic cross-matrix differences, which provides a more informative assessment than correlation alone. Finally, the analytical workflow incorporated explicit metabolite-level and sample-level missingness filtering, improving robustness while avoiding unnecessary *a priori* exclusion of analytes that could still prove informative.

The study also has limitations. Sample size was modest, particularly for the exploratory health comparisons. This is important for the multivariate analyses because PCA patterns and PERMANOVA results may be unstable, underpowered, and sensitive to group imbalance when based on small numbers of dogs relative to the number of measured metabolites. The urine cohort was sampled at a different timepoint from the baseline blood-matrix cohort, which limits direct comparison of matrix performance across all four biospecimens. In addition, the assay covered a targeted subset of small polar metabolites and does not inform comparability for other metabolite classes, particularly lipids. Finally, because the results are platform- and protocol-specific, they should not be generalized uncritically to other anticoagulants, extraction workflows, or analytical platforms.

### 4.6 Practical implications and conclusions

The present findings support serum as the preferred matrix when the goal is to detect diet-associated variation in canine targeted metabolomics of small polar metabolites. Lithium-heparin plasma was globally more similar to serum than EDTA plasma was and may represent an acceptable alternative for selected metabolites, but this should be established on a metabolite-specific basis. EDTA plasma showed greater global divergence from serum, and urine should be regarded as a distinct complementary matrix rather than a blood substitute. More broadly, these results support a conservative recommendation for canine targeted metabolomics: biospecimens should not be pooled or treated as interchangeable unless metabolite-specific comparability has been demonstrated. At minimum, the specific plasma anticoagulant should always be stated, and matrix choice should be aligned with the biological question under investigation.

## Data Availability

The original contributions presented in the study are included in the article/Supplementary material, further inquiries can be directed to the corresponding author.
