# Supplementary material for: Differences among canine serum, plasma and urine metabolite profiles from samples that were collected at the same time
Source: Front Vet Sci. 2026 May 21;13:1837846. doi: 10.3389/fvets.2026.1837846 (PMC13233384; doi:10.3389/fvets.2026.1837846)
Supplement: Supplementary file 1 [file Supplementary_File_1.docx]

Supplementary Material

# Supplementary Tables

Table S1. Overview of the surrogate-label criteria used in the biofluid comparisons.

| **Surrogate label** | **Data requirement** | **Statistic used** | **Decision rule** | **Interpretation** |
| --- | --- | --- | --- | --- |
| insufficient_data | n_pairs below threshold OR is.na(r_pearson) | Pearson correlation on paired log10 concentrations | Dataset 1: n_pairs < 4 → insufficient; Dataset 2 (serum–urine): n_pairs < 3 → insufficient | Not enough paired data to classify reliably. |
| Excellent surrogate | Meets data requirement | r_pearson, q_pearson | r_pearson ≥ 0.90 and q_pearson < 0.05 | Very strong cross-matrix tracking; values move together across matrices. |
| Good surrogate | Meets data requirement | r_pearson, q_pearson | 0.80 ≤ r_pearson < 0.90 and q_pearson < 0.05 | Strong cross-matrix tracking; generally suitable as a surrogate. |
| Moderate surrogate | Meets data requirement | r_pearson | 0.60 ≤ r_pearson < 0.80 | Moderate tracking; use cautiously (especially if bias is non-negligible). |
| Poor surrogate | Meets data requirement | r_pearson | Otherwise | Weak/unstable tracking; do not treat matrices as interchangeable for this metabolite. |

# Supplementary Figures


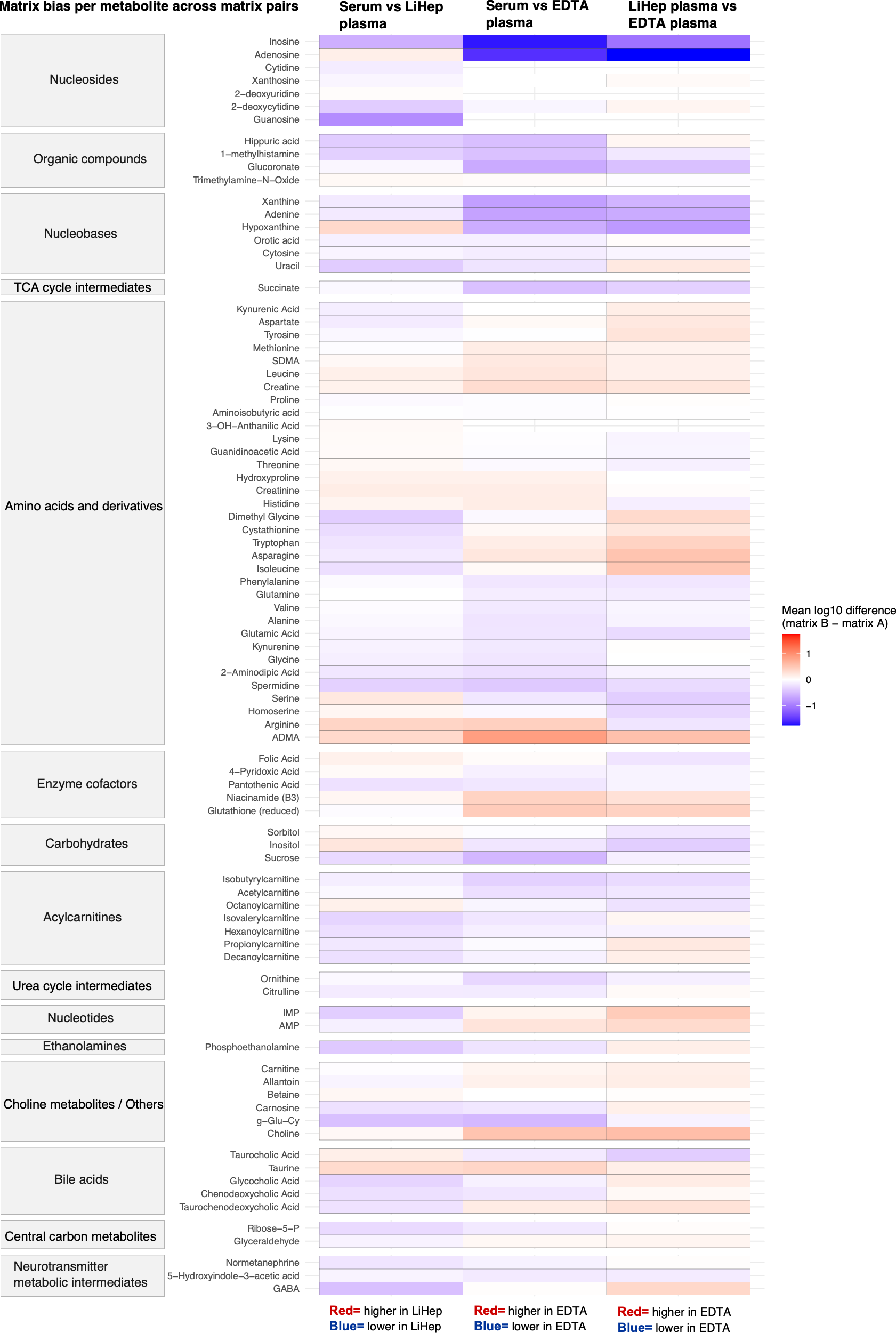


Figure S1. Heatmap showing relative concentration differences across the blood-based matrix pairs, grouped by metabolite class and clustered to maximize within-class contrast.


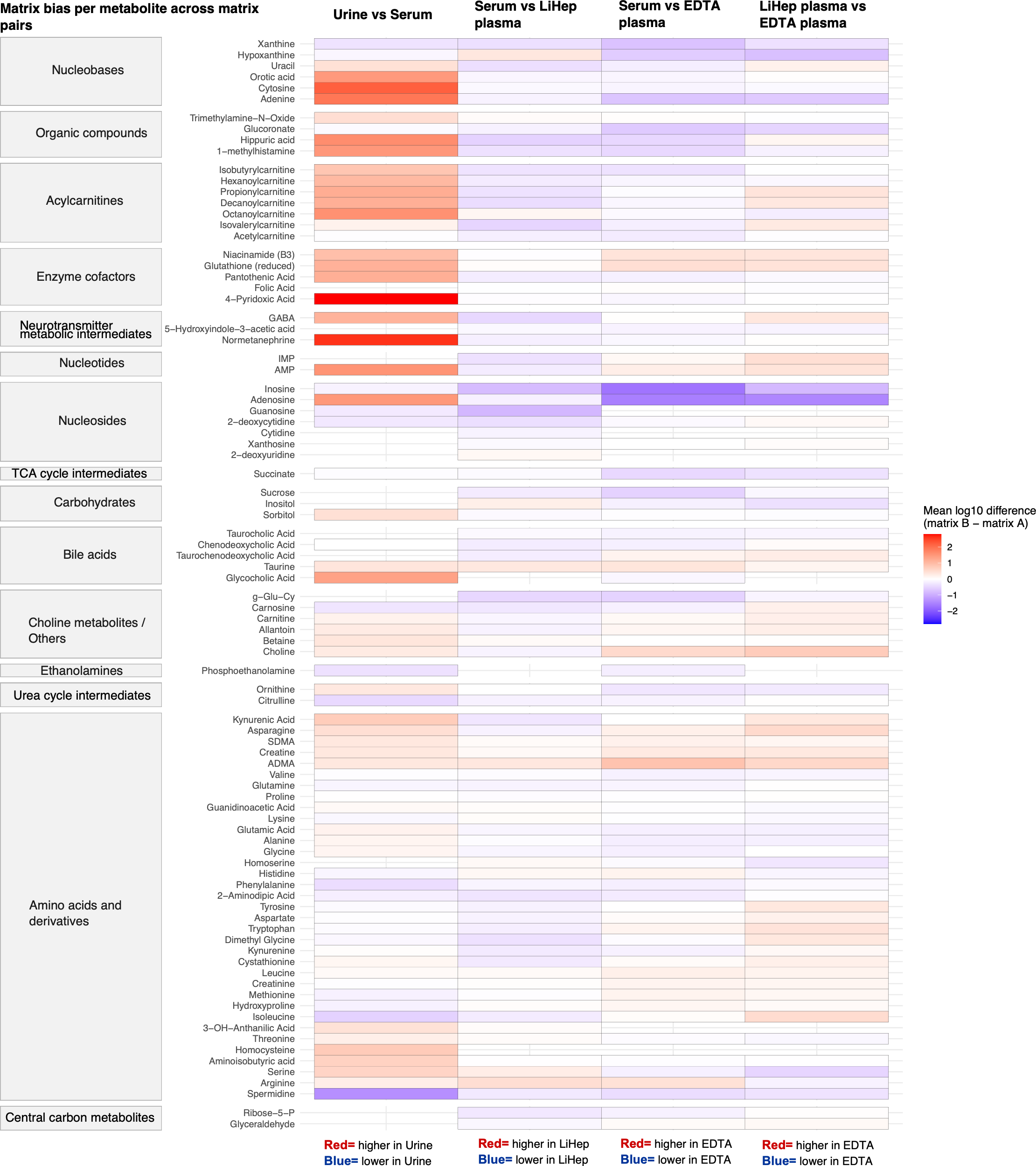


Figure S2. Heatmap showing relative concentration differences across all biofluid pairs, grouped by metabolite class and clustered to maximize within-class contrast.


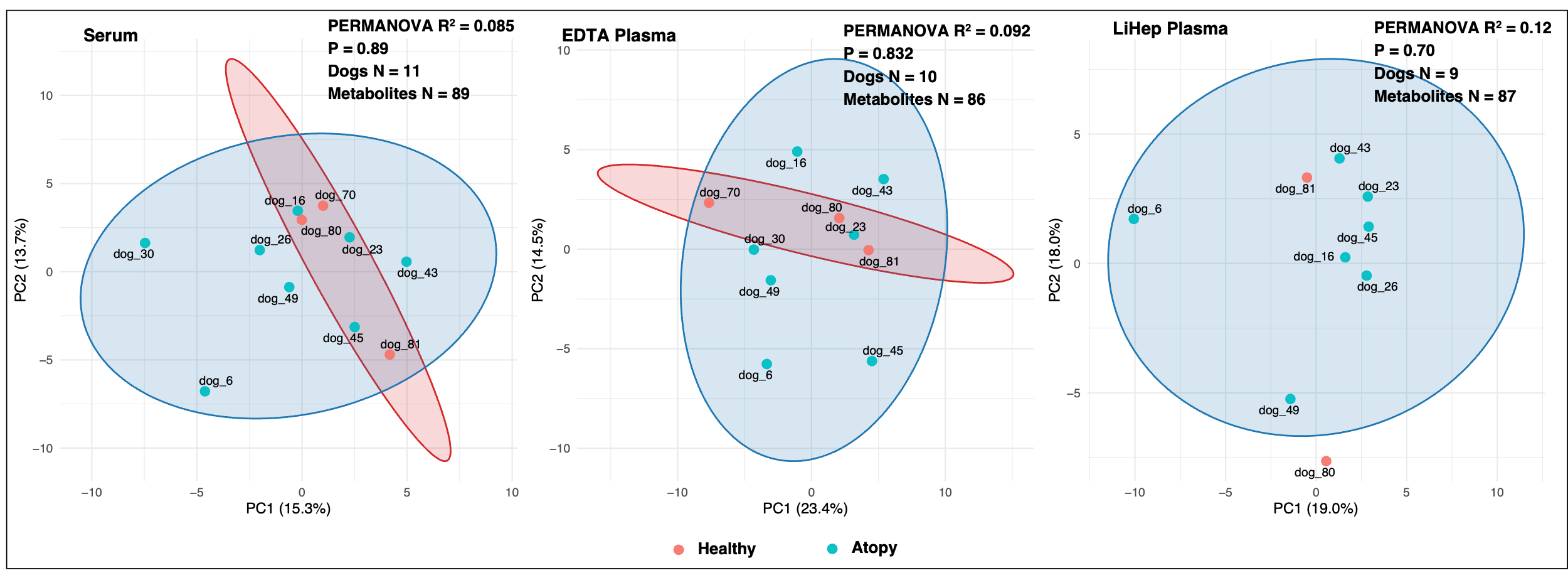


**Figure S3.** PCA plots showing exploratory health-associated separation of metabolite profiles at baseline in serum and EDTA plasma. Panel annotations report PERMANOVA pseudo-R², permutation p-value, number of dogs, and number of metabolites included. Lithium-heparin plasma was not interpreted because too few healthy control samples were available.
